# Application of Dental Pulp Stem Cells for Bone and Neural Tissue Regeneration in Oral and Maxillofacial Region

**DOI:** 10.1155/2023/2026572

**Published:** 2023-03-29

**Authors:** Yasuyuki Fujii, Ayano Hatori, Daichi Chikazu, Toru Ogasawara

**Affiliations:** ^1^Department of Oral and Maxillofacial Surgery, Tokyo Medical University, 6-7-1 Nishishinjuku, Shinjuku-ku, Tokyo, Japan; ^2^Department of Oral and Maxillofacial Surgery, Graduate School of Medicine and Faculty of Medicine, The University of Tokyo, Hongo 7-3-1, Bunkyo-ku, Tokyo 113-8655, Japan

## Abstract

In the oral and maxillofacial region, the treatment of severe bone defects, caused by fractures, cancers, congenital abnormalities, etc., remains a great challenge. In addition, neurological disorders are frequently accompanied by these bone defects or the treatments for them. Therefore, novel bone regenerative techniques and methods to repair nerve injury are eagerly sought. Among them, strategies using dental pulp stem cells (DPSCs) are promising options. Human DPSCs can be collected easily from extracted teeth and are now considered a type of mesenchymal stem cell with higher clonogenic and proliferative potential. DPSCs have been getting attention as a cell source for bone and nerve regeneration. In this article, we reviewed the latest studies on osteogenic or neural differentiation of DPSCs as well as bone or neural regeneration methods using DPSCs and discussed the potential of DPSCs for bone and nerve tissue regeneration.

## 1. Introduction

In the oral and maxillofacial region, bone defects caused by fractures, cancers, or congenital abnormalities result in not only functional disabilities but also cosmetic disturbances. Neurological disorders such as lingual nerve injury, mental nerve paresthesia, and facial nerve paralysis are also caused by third molar extraction, orthognathic surgeries, and benign or malignant tumor surgeries. The psychological suffering of patients with such disorders in the oral and maxillofacial region is unfathomable.

To repair these defects or recover lost functions, reconstructive surgeries for bone or nerve tissue have been performed. However, procedures such as autografts (e.g., iliac crest graft or fibula graft) entail additional surgical stress, and there is a limitation on the amount of tissue that can be grafted. Allograft or xenograft can overcome these problems, but the efficiency of bone regeneration is lower in those procedures than in autografts. There are also a number of methods to repair nerve injury, such as external decompression, direct suture/neurorrhaphy, and autogenous grafts [1]. However, these reconstructive methods do not achieve full recovery, and patients sometimes exhibit limited sensory impairment. Recently, polyglycolic acid sleeves and absorbable collagen sleeves have achieved functional sensory recovery for inferior alveolar nerve and lingual nerve injuries in the oral and maxillofacial region [2]. These sleeves repair small defects of the nerve. On the other hand, the reconstruction of extensive nerve injuries using artificial materials involves a lot of challenges that need to be resolved.

Mesenchymal stem cells (MSCs) are known to be a useful cell source for regenerative therapy. Many types of MSCs, such as bone marrow stromal stem cells (BMSCs) and adipose tissue-derived stem cells (ASCs), have been reported for bone or nerve tissue regeneration [3–13]. Among them, dental pulp stem cells (DPSCs) have been considered a promising cell source for regenerative medicine and tissue engineering. DPSCs, which were first identified in 2000, can differentiate into multiple-lineage cells such as adipogenic, neurogenic, and osteogenic cells [14, 15]. DPSCs demonstrate higher clonogenic and proliferative potential than BMSCs [14, 15]. In addition, DPSCs are very easily isolated from extracted teeth in a less-invasive manner without any ethical issues. Thus, tissue engineering techniques using DPSCs must be established in order to overcome current tissue reconstruction limitations in the oral and maxillofacial region. Herein, we reviewed the advancement of DPSC applications for bone and nerve tissue regeneration and discussed the potential of DPSCs as a target of bone and neural regenerative medicine.

## 2. Bone Regeneration of DPSCs

**Figure 1:**
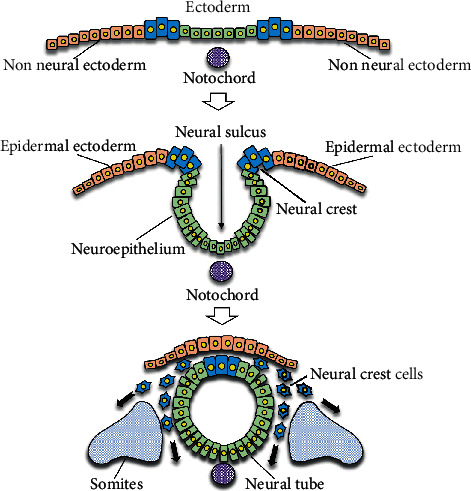
Neural crest formation. DPSCs are derived from neural crest cells.

Craniofacial bones originate from the neural crest, which displays different osteogenic differentiation traits than mesoderm for long bones [16, 17]. A previous study indicated that neural crest-derived progenitors displayed more efficient regeneration of bone tissue than their mesoderm-derived counterparts [18]. Therefore, neural crest-derived MSCs, such as DPSCs, may be suitable for bone regeneration therapy in the oral and maxillofacial region [13, 19] (Figure 1). Many studies have reported that DPSCs have osteogenic/odontogenic differentiation ability. However, some problems remain to be overcome in applying DPSCs in a clinical setting. For example, there are individual differences in the capacity of osteogenic differentiation of DPSCs [20, 21], and both the proliferation and differentiation potential of MSCs including DPSCs also decline with age and after repeated passage [22]. Moreover, the number of cells obtained from dental pulp is limited because physiological secondary dentin and pathological tertiary dentin are formed by odontoblasts with age, resulting in a decrease in pulp tissue volumes [23]. For clinical application, efficient and stable culture methods for osteogenic differentiation of DPSCs or transplantation strategies combining DPSCs and scaffolds are required. In order to establish them, many studies have reported on the molecular mechanism of osteogenic differentiation or bone regeneration using DPSCs. Here, we summarized the recent *in vitro* and *in vivo* studies on osteogenic differentiation of DPSCs or osteogenesis using them, focusing especially on growth factors or small molecules as osteogenic factors, gene engineering, scaffolds, and culture conditions.

### 2.1. Growth Factors, Small Molecules

Many studies have found that recombinant proteins promote the differentiation ability of many types of stem cells. Osteogenic differentiation of DPSCs also can be induced by various growth factors. For example, the bone-healing potential of DPSCs was even stronger when the cells were primed with fibroblast growth factor 2 [24]. PTH (PTH, amino acid 1–34, known as teriparatide), which regulates serum calcium levels and affects bone development, enhances the osteo-/odontogenic differentiation capacity of DPSCs via the ERK and P38 signaling pathways [25]. In addition, bone morphogenetic protein-7 (BMP-7), which belongs to the transforming growth factor-*β* (TGF-*β*) superfamily, is capable of inducing DPSCs toward odontogenic differentiation at appropriate concentration ranges [26]. Insulin-like growth factor-1 (IGF-1) is a multifunctional peptide that plays an important role in bone formation and mineralization [27]. IGF-1 activated mTOR through the PI3K/Akt pathway to induce the differentiation of DPSCs into osteoblasts [28].

Although recombinant proteins are useful for cell proliferation or differentiation, their high cost and instability may require more stable and economical culture methods for clinical application. To overcome these problems, many studies have shown that small chemical compounds induce bone formation. We previously reported that the helioxanthin derivative 4-(4-methoxyphenyl)pyrido[40,30:4,5]thieno[2,3-b]pyridine-2-carboxamide (TH), an osteogenic small molecule, induced the osteogenic differentiation of DPSCs [20, 21, 29]. Moreover, TH successfully promoted the osteogenesis of DPSCs derived from elderly patients, although the proliferation and differentiation potential of MSCs also declines with age and after repeated passage [29–31]. Other small molecules have also been reported as osteogenic molecules for DPSCs. For example, the small molecule compound metformin, a medicine used to treat type 2 diabetes, can induce osteo-/odontogenic differentiation of DPSCs in an AMPK-dependent manner [32]. However, the clinical application of metformin to tissue regeneration is limited because of its rapid dilution. To overcome this disadvantage, a drug delivery system with a controlled release of metformin was developed by using calcium phosphate cement containing chitosan [33]. Ferutinin is a naturally occurring nonsteroid estrogenic compound and has been shown to prevent bone loss in ovariectomized rats [34]. Ferutinin also promotes the osteogenic differentiation of DPSCs by activating the canonical Wnt/*β*-catenin signaling pathway [35]. Aspirin, which inhibits cyclooxygenase and decreases the production of prostaglandins, was reported to promote osteogenic differentiation of bone marrow-derived stem cells by targeting their telomerase activity and to inhibit osteoclast activity in mice [36]. Aspirin also improved the potential of osteogenic differentiation of DPSCs *in vitro* and *in vivo* [37]. Thus, we may be able to establish a novel bone regeneration strategy by searching for osteogenic molecules for DPSCs and their optimal conditions for the osteogenic differentiation of DPSCs.

### 2.2. Gene Engineering

Previous studies have reported that gene engineering enhances the osteogenic potential of DPSCs. For example, transforming growth factor-beta 1 (TGF-*β*1) gene transfer into DPSCs increased osteogenic and chondrogenic differentiation but decreased adipogenic differentiation [38]. In addition, DPSCs overexpressing Sirtuin-1 (SIRT1), an NAD^+^-dependent class III histone deacetylase, promoted bone formation during distraction osteogenesis in a rabbit model [39]. Integrin-*α*5 activity is related to cell proliferation, differentiation, migration, and organ development, and Integrin-*α*5 shRNA promoted the odontogenic differentiation of DPSCs with the enhanced formation of mineralized nodules [40].

MicroRNAs (miRNAs) also play key roles in regulating the osteogenic differentiation of DPSCs. For example, miR-140-5p, which regulates the proliferation and differentiation of cells, is related to the odontoblast differentiation of DPSCs via the Wnt1/*β*-catenin signaling pathway [41]. In addition, the silencing of miR-143-5p promoted the hDPSC odontoblastic differentiation through the activation of the p38 MAPK signaling pathway by upregulating MAPK14 [42]. let-7c-5p, a let-7 miR family member that participates in the regulation of cell proliferation, differentiation, and oncogenesis, was reported to be suppressed in inflamed human dental pulps [43]. Yuan et al. also showed that the induced expression of let-7c-5p could suppress the inflammatory phenomena and restore the osteogenic differentiation potential of inflamed DPSCs [44]. miR-488 suppressed the proliferation and induced the apoptosis of cancer cells [45].

Long noncoding RNAs (lncRNAs) are emerging as important molecules in the odontogenic differentiation of DPSCs. lncRNA H19, one of the most classical lncRNAs, induces the odontogenic differentiation of DPSCs via the H19/S-adenosylhomocysteine hydrolase (SAHH) axis and epigenetically regulates the distal-less homeobox (DLX3) [46]. Thus, the controlled delivery of an osteogenic gene into DPSCs might be a strategy for treating the osteogenesis of DPSCs, although nucleic acids carry the risks of inducing immune responses and genetic alterations through the integration of exogenous sequences into the genome.

### 2.3. Scaffold

These days, there are various scaffolds for the treatment of bone defects in the jaws. Scaffolds with osteoinductive potential for DPSCs have been reported. Collagen is one of the most widely used materials for scaffolds in tissue engineering due to these excellent properties. DPSC-loaded dense collagen gel scaffolds improved bone healing in a rat critical-size calvarial defect model [47]. In addition, *β*-glycerophosphate is a critical factor during the osteogenic differentiation process, and *β*-glycerophosphate-loaded polycaprolactone/polyethylene oxide blend nanofibers demonstrated the osteogenic induction of DPSCs [48]. Platelet concentrates, such as platelet-rich plasma (PRP), platelet-rich fibrin (PRF), and concentrated growth factor (CGF), are also attractive autologous scaffolds suitable for regenerative medicine owing to their fiber architectures and rich growth factors [49, 50]. GCF not only possesses unique fibrin networks and cytocompatibility as a scaffold but it also extracts stimulated cell proliferation, mineralization formation, and odontoblastic differentiation of DPSCs [51]. Several studies have demonstrated that a composite biomaterial including fibrin glue can show increased osteoconductivity and biocompatibility [52]. Nanohydroxy apatite/chitosan/gelatin scaffolds enriched by a combination of platelet-rich plasma and fibrin glue increased the mineralization and osteoblastic differentiation of DPSCs [53]. Mineral trioxide aggregate (MTA) is widely used as a pulp-capping material. MTA is also used for pulp regenerative treatment because it activates cementoblasts and promotes the formation of cementum [54]. Odontogenic differentiation of DPSCs was also induced in MTA, which served as a scaffold in the presence of an odontogenic medium [55]. Gelatin sponges, which are highly biodegradable and have good biocompatibility, are often used for hemostasis. Fu et al. added poly-L-lysine, CaCl_2_, and NaHPO_4_ to gelatin sponges and successfully induced the osteogenic differentiation of DPSCs in this modified gelatin scaffold [56].

On the other hand, scaffold-free transplantation of DPSCs is useful for bone regeneration. A 3D cell culture using cell sheet technology successfully promoted the osteogenic induction of DPSCs [57]. In addition, our previous study showed that DPSC sheets promoted bone regeneration in a bone defect model [20]. We also reported that DPSC sheets facilitated bone formation in fracture sites [21]. Therefore, DPSC sheets may be useful for safe clinical application because infections or immune response after scaffold implantation must be treated.

### 2.4. Cell Culture Conditions

Cell culture conditions affect the properties of DPSCs. Dense culture conditions enhance the osteogenic differentiation of DPSCs via integrin signaling [58]. The conditioned medium of calcined tooth powder, which is obtained from teeth calcined at high temperature (300°C), promotes the osteo-/odontogenic differentiation of DPSCs by triggering MAPK signaling pathways [59]. Moreover, the osteogenic medium for DPSC culture may be improved for clinical application. Supplementation of adenosine triphosphate (ATP) to an osteogenic medium enhanced the osteoblast formation of DPSCs [60]. ATP stimulation increases intracellular Ca^2+^ and mediates intracellular Ca^2+^ signaling via the PLC–IP3 pathway and is initiated by ER release followed by an influx from the extracellular space. Nitric oxide (NO) acts as a biological regulator under both physiological and pathological conditions, and synthetic NO-releasing compounds, called NO donors, induce DPSCs via the TNF*α*-NF-*κ*B axis to differentiate into mature odontoblasts [61]. Low power light (LPL) treatment, which has been known to reduce pain and inflammation and to promote wound healing, also affects the differentiation ability of DPSCs. It was reported that the very low power light at 810 nm enhanced the significant differentiation of hDPSCs in the pulsed wave mode [62].

Different methods of isolating cells from pulp tissue give rise to different populations of DPSCs. The type of enzyme affects the characteristics and behavior of isolated cells. Previously, DPSCs were isolated from pulp tissue by a digestion method using collagenase/dispase or collagenase only. Compared with collagenase I only, DPSCs obtained by collagenase I/dispase treatment had significantly higher numbers of CD146^+^ cells, and the ability of osteogenic/chondrogenic differentiation of DPSCs was higher when collagenase/dispase was used [63].

## 3. Neural Regeneration of DPSCs

Treatment of neuropathies such as spinal cord injury (SCI) includes methods of properly reactivating neural circuits, wiring damaged neural circuits, and repairing neural secondary damage. These treatments can be improved not only by directly regenerating damaged tissue but also by changing the roles of nerves around the site of injury. Some studies reported that methods using scaffolds for cell transplantation and neural circuit reformation can be expected to repair nerves at the injured site [64]. MSCs such as neural stem cells (NSCs) are used for this cell transplantation [65]. Among NSCs, DPSCs that originate from a neural crest origin may have a special potential for neural differentiation, because DPSCs express several neural markers, such as Nestin (neuroectodermal stem cell intermediate filament marker), *β*-3 tubulin (Tuj1), neurotrophin receptors, and neurofilaments [66]. Moreover, some studies differentiated DPSCs into neural-like cells *in vitro* and *in vivo* [67]. DPSCs also express pluripotency-related core factors such as Oct4, Sox2, and Nanog, as well as neural crest markers like Snail, Slugs, Sox10, and HNK1 [68]. Thus, DPSCs have a higher potential for neural cell lineages as well as stemness and are considered a suitable cell source for neural regeneration.

Axon regeneration is divided into the peripheral and central nervous systems. NSCs for cell-based therapies are among the most important therapies for the central and peripheral nervous systems. NSCs derived from brain parenchymal and olfactory bulb can give rise to both neurons and glial cells [69]. However, NSCs present some problems in that the collection from neurosurgical procedures is highly invasive and production is inefficient. Therefore, NSC-based therapies lack versatility. On the other hand, DPSCs are easily obtained from extracted teeth, such as wisdom teeth, by less-invasive surgery and present no ethical issues. Also, Sakai et al. reported that stem cells from human exfoliated deciduous teeth (SHED) transplanted into the severed spinal cord preserve the myelin sheath and differentiate into mature oligodendrocytes known as the central nervous system myelin sheath [70]. In addition, DPSCs may be a candidate cell source for peripheral nerve regeneration because DPSCs support nerve cell growth and can differentiate into Schwann-like cells *in vitro* [71]. Schwann cells derived from DPSCs have been shown to help guide peripheral axonal elongation and myelination *in vitro* [71]. Moreover, DPSCs differentiated into neuronal cells have characteristics similar to those of peripheral neurons compared to peripheral nervous system- (PNS-) derived neurons [67]. Further, neuron-like cells derived from DPSCs have action potential-dependent sodium and potassium channels and generate action potentials [72]. Therefore, there are increasing expectations for nerve regeneration using DPSCs as a resource for neurodegenerative diseases and nerve defects.

### 3.1. Neural Characteristics and Neurogenic Differentiation of DPSCs

In cell-based therapies, specific cells are transplanted to the lesion site, but it is difficult for them to survive in an unfavorable regenerative microenvironment. These transplanted cells need to adapt to the new environment and participate in the activity of surrounding cells [73]. DPSCs are considered highly versatile in nerve regeneration because they have excellent adaptability to harmful metabolic states and can secrete various neuroprotective and immunomodulatory factors [71, 74, 75]. For example, DPSCs are well known for secreting neuroprotective growth factors such as brain-derived neurotrophic factor (BDNF), nerve growth factor (NGF), glial cell line-derived neurotrophic factor (GDNF), neurotrophin-3 (NT-3), vascular endothelial growth factor (VEGF), and platelet-derived growth factor (PDGF) [74, 76, 77]. Some studies found that DPSCs expressed these neuroprotective growth factors *in vitro* more highly than other MSC types [73, 74, 78, 79]. Secretion of these growth factors can reduce neural apoptosis and neurodegeneration in the early stages of sensory neuron survival [70, 80–82].

MSCs can work as immunosuppressive agents by regulating immune responses such as inflammatory or autoimmune diseases [83]. Among MSCs, DPSCs are also known to secrete powerful immunomodulatory and anti-inflammatory cytokines such as transforming growth factor-beta (TGF-*β*), interleukin-8 (IL-8), interleukin-6 (IL-6), hepatocyte growth factor (HGF), and indoleamine 2,3-dioxygenase (IDO) [84, 85]. In fact, DPSCs have been shown to significantly inhibit the activation of osteoarthritis macrophages *in vitro* and to reduce cell morphology, immune phenotype, and expression of inflammatory factors [86]. DPSC suppressed the activation of osteoarthritic macrophage in osteoarthritis [86]. Also, the monocytic mobilizing protein-1 (MCP-1) and the secretory external domain of sialic acid-binding Ig-like lectin-9 (ED-Siglec-9) secreted from DPSCs promoted recovery after rat SCI [87]. Yan et al. showed that DPSCs and BMMSCs could depress the function of NK cells by hydrolysing ATP to ADO using CD39 and CD73 enzymatic activity [88]. Therefore, it can be suggested that DPSCs have strong immunosuppressive properties that help control inflammation at each site and promote nerve regeneration.

Neurogenic factors such as growth factors or small molecules are necessary to induce DPSCs into neuron-like cells. Many methods for the neurogenic induction of DPSCs depend on growth factors such as basic fibroblast growth factor (bFGF), epidermal growth factor (EGF), NGF [89], BDNF, GDNF, and sonic hedgehog; various small molecules such as NT-3, retinoic acid (RA) [90], forskolin, and heparin; and culture supplements such as B27, insulin-transferrin-sodium selenite (ITS), nonessential amino acids, and N2 [91, 92]. bFGF and NGF exerted a synergistic regulatory effect on the neural differentiation of DPSCs [89]. Additionally, Sugiyama et al. showed that FGF-2-pretreated DPSCs improved recovery in a rat model of spinal cord injury (SCI) [93]. The simultaneous activation of PKC and cAMP induces the differentiation of DPSCs into functionally active neurons [72]. Also, IGF-1 (insulin-like growth factor-1) can enhance the neural differentiation of DPSCs via the activation of the mTOR signaling pathway [94]. The addition of bone morphogenetic protein type 4 (BMP4) to DPSCs cultured as neurospheres improved the NSC characteristics and neural differentiation [95]. Recently, Heng et al. showed that small molecules (valproic acid, CHIR99021, RepSox, forskolin, SP600125, GO6983, Y-27632, and dorsomorphin) can enhance the neurogenic differentiation of iPSCs (induced pluripotent stem cells), SCAPs (stem cells from apical papilla), and GMSCs (gingival mesenchymal stem cells) [96].

The neural differentiation mechanisms of DPSCs were clarified previously. Heng et al. indicated that stimulation of the forward ephrinB2-EphB4 signal markedly inhibited the neurogenesis in DPSCs, whereas suppression of this forward signal pathway with a peptide inhibitor specific to EphB4 accelerated neurogenesis [97]. Moreover, the Nell-1 gene, which is known to promote osteoblast differentiation and dentin formation, promoted the neural-like differentiation of dental pulp cells [98]. Also, exogenous expression of the OLIG2 (oligodendrocyte lineage transcription factor 2) gene could be used as an efficient way to induce the differentiation of DPSCs into functional oligodendrocytes [99]. Recently, Kogo et al. indicated that epigenetic reprogramming along with cell cycle regulation by stimulation with high K^+^ accelerated the differentiation of IPSCs into neuron-like cells [100]. As noted above, a highly efficient DPSC neural differentiation method has been getting attention.

### 3.2. Scaffold and Cell Culture

Application of the appropriate scaffold may improve the proliferation, differentiation, adhesion, and migration of DPSCs. It may repair damaged tissue and promote the ability to regenerate functional organs. Most of the *in vitro* studies were performed using polymers of natural origin such as polysaccharide-based materials, including cellulose and chitosan, and protein-based materials, including collagen, gelatin, silk, fibronectin, and fibrin [101, 102]. For example, granular 3D chitosan scaffolds provide a preferable microenvironment for the proliferation, attachment, and neural differentiation of DPSCs [103]. A decellularized extracellular matrix (ECM) scaffold was shown to affect the differentiation of DPSCs into a neuron-like of a neuron-like organization, including a neurite outgrowth promotion [104]. Luo et al. showed that filling a cellulose/soy protein isolate composite membrane (CSM) hollow conduit with a 10% GFD formula (GelMA-bFGF) hydrogel loaded with DPSCs was effective for nerve regeneration [101]. However, these polymers of natural origin need to be improved because they are of varying quality, have limited mechanical properties, and entail the risk of provoking immune reactions [105].

On the other hand, synthetic biomaterials, such as polymer-based biomaterials (for example, poly-L-lactic acid, polylactic acid-coglycolic acid copolymer, poly(L-lactide-co-6-caprolactone), polyglycolide, and poly-ethylene glycol) and ceramic-based biomaterials (for example, hydroxyapatite, bioactive glass, and calcium phosphate), display better mechanical properties, reproducibility, and electrical conductivity compared to polymers of natural origin [102]. PLGA artificial nerve conduits with DPSCs promoted facial nerve regeneration [106]. Also, reduced graphene oxide- (RGO-) polycaprolactone (PCL) hybrid electrospun NFs enhanced the neurogenesis of DPSCs [107]. Furthermore, the use of bioactive molecules such as growth and angiogenic factors in appropriate scaffolds has been suggested as a promising strategy for improving DPSC transplantation. Luo et al. suggested that transplanted thermosensitive heparin-poloxamer (HP) hydrogel that contained DPSCs and bFGF had a significant impact on spinal cord injury repair and regeneration in rats [108].

In 1992, Reynolds and Weiss announced that the addition of EGF to cells taken from the striatum of adult mice can separate cell clusters that produce nerve cells and glial cells while dividing and proliferating [109], and they called these cell clusters “neurospheres” for the first time. Subsequent research showed that the neurosphere culture method increased the ability of NSCs to maintain the undifferentiated state, and neurons, astrocytes, and oligodendrocytes were produced from the neurospheres by changing the culture conditions [110]. Similar to NSCs, DPSCs under neurosphere conditions tend to form floating or poorly adherent spheroids. These spheroids, also called pulp “dentospheres,” present some shared characteristics with neurospheres [111].

It is necessary to refrain from adding an animal serum to the culture medium for the clinical application of nerve regeneration therapy. Even the temporary presence of xenogeneic serum components in culture conditions can cause unwanted immune responses and even rejection of transplanted cells [112, 113]. Although serum-free culture protocols without FBS have been demonstrated, the growth rate of DPSCs in serum-free medium is usually lower than when animal serum is used [68]. In this situation, we successfully isolated and cultured DPSCs in xeno-/serum-free culture conditions, and we also generated suspended neurospheres from these xeno-/serum-free DPSCs and differentiated them into neurons [113]. More highly efficient neuronal differentiation of DPSCs under xeno-/serum-free conditions will lead to innovation in neurodegenerative cell therapy.

DPSCs have already been investigated for the treatment of neurodegenerative diseases such as neuropathic polyneuropathy, Parkinson's disease, and Alzheimer's disease using the above neurological and immunosuppressive properties [114–116].

These studies indicated that DPSCs may differentiate toward neuronal-like cells that function in the central nerve system (CNS). Besides, some studies suggested that DPSCs improved nerve dysfunction. DPSCs counterbalanced oxidative stress induced by sciatic nerve injury and supraspinal neuroinflammation in rat brain [117]. Tsuruta et al. showed that SHED-CM (SHED-conditioned medium) injected intravenously improved superior laryngeal nerve injury with dysphagia in a rat model [118].

## 4. Conclusion

**Figure 2:**
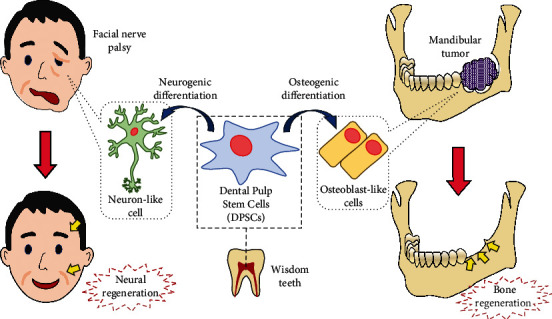
Graphical abstract. A therapeutic strategy for bone and neural regeneration with DPSCs.

In this article, we reviewed osteogenic and neural differentiation and regeneration using DPSCs, and we added 16 vivo studies, 9 of which were articles on bone regeneration [20, 21, 24, 29, 37, 39, 47] and 7 of which were articles on neuroregeneration [93, 101, 106, 108, 114, 116–118]. Several lines of evidence strongly suggest that DPSCs, which are derived from the neural crest, are among the most suitable cell sources for bone or neural regeneration therapy in the oral and maxillofacial region (Figure 2 and Table 1), because craniofacial skeletal tissues mainly have the same origin as DPSCs. Meanwhile, an in-depth understanding of the characteristics and the differentiation mechanism of DPSCs is required for cell-based therapies. In addition, the appropriate use of growth factors, small molecules, gene engineering, or scaffolds in culture conditions or transplantation is indispensable for tissue regeneration using DPSCs. At the same time, we need to consider the safety and stability of these methods for clinical application. Therefore, the establishment of bone and neural regeneration therapies using DPSCs in the oral and maxillofacial region requires both more basic studies and more preclinical and clinical studies.

## Figures and Tables

**Table 1:** Characteristics of DPSCs in comparison with BMSCs, ASCs, and NSCs.

	DPSCs	BMSCs	ASCs	NSCs
Origin	Dental pulp (neural crest)	Bone marrow (mesoderm)	Adipose tissue (mesoderm)	Brain, olfactory bulb (ectoderm)

Isolation method	Tooth extraction (no additional invasive treatment)	Painful process (bone marrow aspiration)	Easily accessible procedure (liposuction)	Neurosurgical procedures (obtained from brain parenchymal)

Isolation quantity	Limited volume (decrease pulp tissue volumes with age [23])	Low cell yields	Large quantity	Limited volume (high risk and ethical issue)

Pluripotency	Multilineage differentiation (e.g., osteogenic, adipogenic, and neurogenic differentiation) [12, 14, 15]			Neural cells types (neurons, astrocytes, and oligodendrocytes) [69]

Proliferative potential	DPSCs>BMSCs [14, 15] DPSCs>ASCs [8]			

Osteogenic differentiation	DPSCs < BMSCs [5, 6] DPSCs >BMSCs [7, 19] BMSCs>ASCs [9, 10, 11] DPSCs < ASCs [8]			

Craniofacial bone regeneration	DPSCs may be suitable for bone regeneration therapy in the oral and maxillofacial region? DPSCs=BMSCs [13] DPSCs > BMSCs [19]			

Neurogenic differentiation	Neuroprotection and neurogenesis: DPSCs>BMSCs>ASCs [73, 74, 78, 79] Secretion of neurotrophic factors: DPSCs>BMSCs [74, 77, 78] Secretion of grows factors: DPSCs >BMSCs=ASCs [78, 82] Immunomodulatory and anti-inflammatory cytokines DPSCs >BMSCs [74, 85]			

Neural markers expression	Progenitor markers NSCs=DPSCs [66, 67] Mature markers NSCs > DPSCs [68, 71]			

## Data Availability

No underlying data was collected or produced in this study
